# The Role of AI-Driven De Novo Protein Design in the Exploration of the Protein Functional Universe

**DOI:** 10.3390/biology14091268

**Published:** 2025-09-15

**Authors:** Guohao Zhang, Chuanyang Liu, Jiajie Lu, Shaowei Zhang, Lingyun Zhu

**Affiliations:** College of Science, National University of Defense Technology, Changsha 410073, China; zhangguohao@nudt.edu.cn (G.Z.); liuchuanyang13@nudt.edu.cn (C.L.); lujiajie21@nudt.edu.cn (J.L.)

**Keywords:** de novo protein design, protein functional universe, AI-driven toolkit

## Abstract

The extraordinary diversity of protein sequences and structures gives rise to a vast protein functional universe with extensive biotechnological potential. Nevertheless, this universe remains largely unexplored, constrained by the limitations of natural evolution and conventional protein engineering. Substantial evidence further indicates that the known natural fold space is approaching saturation, with novel folds rarely emerging. AI-driven de novo protein design is overcoming these constraints by enabling the computational creation of proteins with customized folds and functions. This review systematically surveys the rapidly advancing field of AI-based de novo protein design, reviewing current methodologies and examining how cutting-edge computational frameworks accelerate discovery through three complementary vectors: (1) exploring novel folds and topologies; (2) designing functional sites de novo; (3) exploring sequence–structure–function landscapes. We highlight key applications across therapeutic, catalytic, and synthetic biology and discuss the persistent challenges. By fusing recent progress and the existing limitations, this review outlines how AI is not only accelerating the exploration of the protein functional universe but also fundamentally expanding the possibilities within protein engineering, paving the way for bespoke biomolecules with tailored functionalities.

## Simple Summary

Proteins are the molecular machines of life, essential for countless processes from building cellular structures to fighting disease. Nature has created a stunning array of these molecules, but this diversity is merely a glimpse of what is theoretically possible. This vast, untapped potential holds promise for solutions to some of our biggest challenges, such as cleaning up pollution, curing diseases, and creating new materials. Exploring these possibilities by experiments alone is impossibly slow and expensive. This review explains how artificial intelligence (AI) is changing the game. AI now allows us to design entirely new proteins de novo on a computer, predicting how they will fold and function. This powerful approach is yielding breakthroughs across biotechnology at an unprecedented pace. As AI continues to evolve, it promises to unlock a new era of biological engineering, providing custom-made protein tools for advances in medicine, agriculture, and green technology.

## 1. Introduction

Artificial intelligence (AI) is causing a paradigm shift across numerous fields within biology and medicine [1,2]. In particular, AI-driven approaches are increasingly being harnessed to tackle one of the most fundamental challenges in protein engineering: the exploration and design of functional proteins [3]. Proteins are central to virtually all biological processes, yet the vast majority of possible protein sequences and structures remain unexplored, constrained by evolutionary history and by experimental throughput.

Conventional protein engineering, while yielding remarkable successes, is inherently limited by its dependence on existing biological templates. Current methods often fail to access novel functional regions of the protein universe that lie beyond natural evolutionary pathways. Moreover, they typically require experimental screening of large variant libraries, a process that is labor-intensive, costly, and ultimately confined to incremental improvements within well-explored neighborhoods of the sequence–structure space [4,5]. Consequently, systematic exploration of the uncharted territories within the protein functional universe demands a disruptive, more pioneering approach.

Recent advances in AI, however, have begun to transcend these limitations. By integrating generative models, structure prediction tools, and iterative experimental validation, AI-driven de novo protein design offers a powerful framework for systematically exploring and engineering proteins with customized functions. This approach leverages known statistical patterns from vast biological datasets to establish high-dimensional mappings between sequence, structure, and function, enabling the rapid generation of novel, stable, and functional proteins. This approach empowers researchers to directly explore regions of the functional landscape that natural evolution has not sampled, thereby accelerating the discovery of novel biomolecules and opening new avenues for addressing global challenges in health, sustainability, and biotechnology.

## 2. The Vast but Evolutionarily Constrained Protein Functional Universe

**Figure 1:**
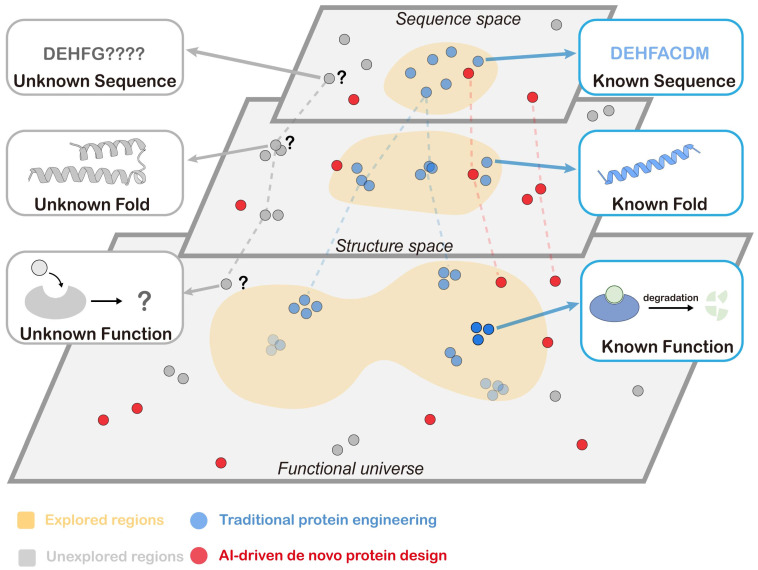
Schematic of the protein functional universe. The vast, high-dimensional mapping between sequence and structure spaces defines an immense protein functional universe. The yellow region denotes the sequence/structure/function space already explored via natural evolution and experimental characterization; the shaded gray region denotes largely unexplored regions of the sequence/structure/functional space. Each circle represents an individual protein characterized by its sequence, structure, and function. Red circles indicate proteins discovered and characterized via AI-driven de novo protein design; blue circles indicate proteins obtained and characterized through traditional protein engineering or evolutionary methods; gray circles indicate sequences/structures/functions that remain unknown or uncharacterized. Traditional engineering and evolution largely sample the well-explored neighborhood, whereas AI-driven de novo design can systematically probe and populate distant, previously inaccessible regions, enabling the discovery of novel sequences, folds, and functions.

Proteins drive critical cellular processes, including enzymatic catalysis [6,7], signal transduction [8,9,10], molecular recognition [11,12], structural support [13,14,15], and immune defense [16,17], highlighting their extensive functional diversity. This breadth of activity constitutes the “protein functional universe”: a theoretical space encompassing all possible protein sequences and structures and the biological activities they can perform, governed by the complex mapping between sequence space and structure space. This conceptual universe includes not only the folds and functions observed in nature but also every other stable protein fold and corresponding activity that could in principle exist (Figure 1). Systematically probing the unexplored functional universe could reveal novel enzymes, binding activities, or molecular machines that do not exist in the natural world, opening up new solutions in biotechnology, medicine, and synthetic biology [18].

Researchers exploring this universe, however, face two fundamental challenges. The first is the problem of combinatorial explosion, stemming from its unimaginable scale. The sequence → structure → function paradigm—the idea that a protein’s amino acid sequence encodes its three-dimensional fold, which in turn largely determines its biological function—is a longstanding central tenet of molecular biology [19,20]. For perspective, a mere 100-residue protein theoretically permits 20^100^ (≈1.27 × 10^130^) possible amino acid arrangements, exceeding the estimated number of atoms in the observable universe (~10^80^) by more than fifty orders of magnitude [21]. This renders the prior probability that a random sequence will fold stably and display useful activity vanishingly small. Given that experimental investigations are restricted and costly, unguided experimental screening is profoundly inefficient [22].

The second challenge arises from the constraints of natural evolution. Despite their functional richness, natural proteins are products of evolutionary pressures for biological fitness, and are not necessarily optimized as versatile tools for human utility. This so-called “evolutionary myopia” tends to lead to proteins that are optimized for survival in specific niches, potentially limiting properties such as stability, specificity, or suitability for industrial conditions. Comparative analyses suggest that known protein functions represent only a tiny subset of the diversity nature can produce [23]. Furthermore, the current evidence suggests that the known protein fold space may be nearing saturation, with recent functional innovations predominantly arising from domain rearrangements [24,25]. Integrating predicted structures reveals how environmental pressures profoundly shape natural protein diversity: evolutionary forces overwhelmingly favor domain recombination over de novo emergence of structural motifs or folds. Coupled with the accumulation of gradual genetic variation in natural populations, this selective paradigm reinforces an evolutionary trajectory that diversifies proteomes through reorganization and repurposing—thereby constraining the exploration of genuinely novel sequences and structures [24].

Despite considerable advances in mapping the existing sequence and structure spaces—exemplified by resources such as the MGnify Protein Database [26] (cataloging nearly 2.4 billion non-redundant sequences) and the Profluent Protein Atlas v1 [27] (encompassing over 3.4 billion full-length proteins)—alongside expanding structural repositories (including ~214 million models in the AlphaFold Protein Structure Database [28] and about 600 million predicted structures in the ESM Metagenomic Atlas [29], these datasets constitute an infinitesimally small portion within the theoretical protein functional space. Public datasets are also biased by evolutionary history and assay ability, which tends to channel data-driven methods toward well-explored regions of the sequence–structure space. Thus, vast regions of the sequence–structure space remain inaccessible, highlighting the need for a new paradigm that integrates advanced computation with experimental validation to unlock the immense latent functional potential within the uncharted protein universe [22,30,31].

## 3. Beyond Evolutionary Boundaries: Exploring the Functional Universe

This imperative for a new approach is underscored by the intrinsic limitations of conventional protein engineering strategies. While methods such as directed evolution have proven powerful for optimizing existing proteins [4,5], their workflow inherently constrains exploration. By necessitating a natural protein as a starting point, they remain tethered to evolutionary pressure and the requirements for the construction and experimental screening of immense variant libraries through iterative cycles of mutation and selection. This is not just labor-intensive and costly; more fundamentally, it confines discovery to the immediate “functional neighborhood” of the parent scaffold, performing a local search within the vastness of the protein functional universe. Consequently, these approaches are structurally biased and ill-equipped to access genuinely novel functional regions that lie beyond the boundaries of natural evolutionary pathways [32,33,34].

De novo protein design aims to transcend these limits by designing proteins from first principles to meet specified structural or functional objectives, rather than by modifying existing scaffolds [32]. This approach can, in principle, produce wholly new folds, bespoke active sites, and modular components with engineered properties (tunability, controllability, and modularity), offering a systematic route to functions that natural evolution has not explored [33,35,36,37]. This fundamental paradigm shift frees protein engineering from its historical reliance on natural templates and removes their inherent evolutionary limitations; exploration of the functional protein universe transitions from empirical trial-and-error explorations to systematic rational design, vastly expanding our access to the previously unimaginable diversity of biologically active folds and functions.

However, de novo design itself faces fundamental challenges. Functional proteins occupy an astronomically small subset of the possible sequence–structure space, and as the polypeptide length increases, the conformational freedom, synthesis cost, and assay complexity grow exponentially. The precise mapping between protein sequence and structural attributes and functional phenotypes defines the canonical “fitness landscape” [38]. In practical de novo design, searches must therefore be restricted to computationally tractable subspaces, and precise atomic constraints should be imposed for functional geometries to ensure orthogonality to the host biology and to avoid off-target interactions.

### 3.1. The AI-Driven Paradigm Shift in Protein Engineering

Historically, de novo protein design has relied heavily on empirical methods and physics-based modeling [39,40,41,42]. Rosetta is a typical example, operating on Anfinsen’s hypothesis that proteins fold into their lowest-energy state [43]. By taking a blueprint of secondary structure elements (e.g., α-helices, β-sheets), Rosetta employs fragment assembly and force-field energy minimization to fold proteins in silico and stitches together short peptide fragments from known proteins and performs conformational sampling (e.g., Monte Carlo with simulated annealing). The lowest-energy conformations under its force field are then selected as candidate designs [39]. In 2003, Kuhlman et al. used Rosetta to create Top7, a 93-residue protein with a novel fold not observed in nature [44]. Subsequent work extended Rosetta to design enzyme active sites [45,46] and drug-binding scaffolds [47,48,49], showcasing its versatility in rational protein engineering.

Nevertheless, these physics-based methodologies exhibit inherent drawbacks. Firstly, the underlying force fields retain an approximate character: despite contemporary refinements, accurately computing a protein’s comprehensive energy landscape remains challenging, particularly when incorporating elaborate side-chain packing and solvent effects. Even marginal inaccuracies in energy estimates can yield designs that misfold or fail to achieve intended functionality in vitro. Secondly, the associated computational expense is considerable: exhaustive sampling of even a constrained fraction of the sequence and structure space is frequently infeasible [32]. These constraints are acutely observable for large or structurally complex proteins, limiting both throughput and the practical exploration of distant regions of the protein functional universe.

In response, modern AI-augmented strategies have emerged to complement and extend physics-based design [50,51]. Machine learning (ML) models trained on large-scale biological datasets that can establish high-dimensional mappings learned directly from sequence–structure–function can capture intricate interdependent fitness relationships [52,53]. These learned priors accelerate sampling and scoring, enable scalable generative workflows, and guide experiments by prioritizing candidates with higher a priori chances of success. This capability has been fueled by decades of experimental progress—high-throughput DNA sequencing that has exponentially expanded sequence repositories; structural biology innovations such as cryo-electron microscopy (cryo-EM) that uncover atomic-resolution structures [54]; and deep mutational scanning enabling systematic functional profiling [55]. AI-driven de novo protein design commonly integrates hybrid pipelines—generating backbones or sequences, rescoring with structure predictors and energy models, and iteratively incorporating experimental feedback—to make it both more efficient and more expansive in scope. These strategies do not replace experiments, but they transform them from brute-force searches into focused, data-efficient campaigns that stand a realistic chance of revealing novel, functional proteins.

By partitioning the combinatorial search into tractable subspaces and enforcing target-specific constraints, these pipelines enable focused searches for foldable, functional proteins while also promoting orthogonality with the host biology through designing interaction sites specific to the intended targets through rapid in silico screening (e.g., docking/binding [56,57], epitope and immune-response [58], and solubility prediction [59]).

### 3.2. Main Paradigms of AI-Driven De Novo Protein Design

**Figure 2:**
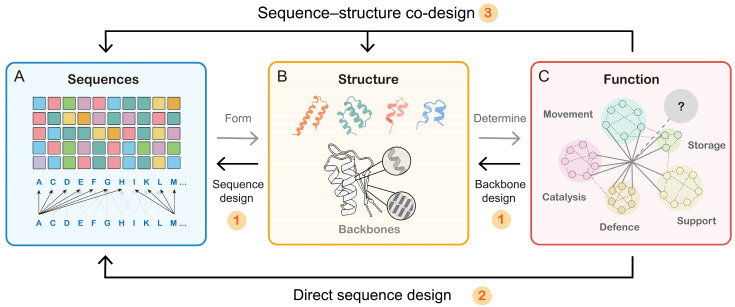
Illustration of the classic sequence → structure → function paradigm and how de novo design inverts it. (**A**) In the classical view, permutations of the 20 amino acids generate a vast sequence space, (**B**) which gives rise to a diverse structural/fold space (**C**) and thereby underpins functional diversity. De novo protein design fundamentally inverts the classic sequence → structure → function paradigm: it starts from a desired function and works backward to derive compatible structures and sequences. Current methodologies fall into three categories: (1) Two-Stage Generative Design, (2) Sequence-Guided Language Methods, and (3) Sequence–Structure Co-Guided Methods.

AI-driven approaches provide a rational, systematic framework for de novo protein design, which can be broadly classified into three main paradigms [33] (Figure 2):

1. Two-Stage Generative Design

Modern AI-driven de novo design typically involves two stages: First, generative models such as diffusion networks construct novel protein backbone geometries or scaffold topologies tailored to specific functional or structural requirements. Next, sequence-design algorithms assign amino acid identities predicted to fold stably into these backbones and exhibit the desired activity.

2. Sequence-Guided Language Methods

Inspired by breakthroughs in natural language processing, large pre-trained protein language models trained solely on sequence data have opened new avenues for de novo protein design by generating and evaluating candidate proteins without explicit structural inputs.

3. Sequence–Structure Co-Guided Methods.

Moreover, based on the fact that protein function arises from intricate sequence–structure interdependencies, recent methods jointly model both structure generation and sequence optimization. These co-design frameworks improve foldability and functional accuracy, leading to higher-confidence de novo protein design.

Collectively, these methodologies advance de novo protein design toward on-demand biomolecular engineering, which would transform drug discovery, synthetic biology, and biomaterials science. AI-driven frameworks transcend natural evolutionary constraints, enabling the de novo design of proteins with novel functions inaccessible via natural evolution. Unified can approaches map multidimensional functional landscapes—from synthetic organelles to biological quantum sensors—effectively rendering the protein universe a programmable platform for medicine, energy, and synthetic biology.

## 4. The AI Toolbox for De Novo Protein Design

**Figure 3:**
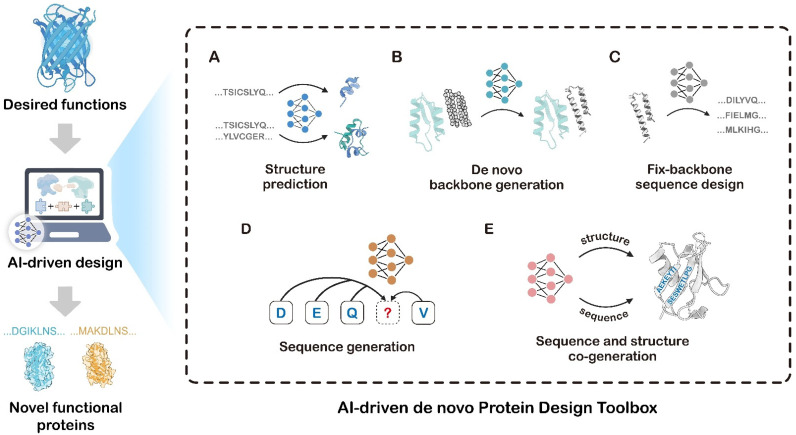
According to their roles in de novo protein design, we categorize the AI-driven de novo protein design toolbox into five classes: (**A**) Protein Structure Prediction models; (**B**) De novo Backbone Generation models; (**C**) “Fixed-backbone” Sequence Design models; (**D**) Sequence Generation models; (**E**) Sequence–structure co-design models.

In practice, AI-driven de novo protein design typically couples generative models with predictive models in an iterative “digital evolution” loop [18]. Predictive tools originally developed for structural determination, such as RoseTTAFold All-Atom [60] and AlphaFold3 (AF3) [56], have become indispensable components of the AI-driven de novo protein design toolbox. These predictive tools provide atomic-precision analyses of target binding sites and employ reverse-engineering of active-site relationships to optimize dynamic stability and functional compatibility [61]. Together with generative models, sequence-design algorithms, and predictive structure tools form a powerful, integrated AI-driven toolbox for de novo protein design (Figure 3).

For readers new to computational protein design, Table 1 summarizes the practical workflows for each toolkit; each row summarizes the goal, the typical inputs, and the typical outputs that beginners can follow as an actionable starting point. The following section provides a concise overview of advancements in the AI-driven de novo protein design toolbox, categorizing its components into five functional classes (Table 2): (i) Protein Structure Prediction (Figure 3A); (ii) De novo Backbone Generation (Figure 3B); (iii) “Fixed-backbone” Sequence Design (Figure 3C); (iv) Sequence Generation (Figure 3D); and (v) Sequence–Structure co-design (Figure 3E).

### 4.1. Protein Structure Prediction

Accurately predicting the three-dimensional (3D) structure of a protein from its primary amino acid sequence has been a longstanding challenge in computational and structural biology [62]. Traditional experimental methods such as nuclear magnetic resonance (NMR) [63], X-ray crystallography [64], and cryo-electron microscopy (cryo-EM) [54] are limited by their low throughput and challenges in capturing dynamic information [65]. AI has transformed this field: at the 14th Critical Assessment of Protein Structure Prediction (CASP14) in 2020, AlphaFold2 (AF2) achieved an atomic-level prediction accuracy comparable to experimental crystallographic resolutions [66,67,68]. AF2 employs coevolutionary signals from multiple sequence alignments (MSAs) within a two-stage framework: the Evoformer network refines evolutionary and geometric constraints, then the structure module assembles backbone coordinates [67]. This breakthrough effectively resolved a decades-long protein-folding enigma and ushered in a transformative era for structural biology. Inspired by AF2, many other groups have developed high-performance prediction models. The Baker laboratory developed RoseTTAFold [69], which uses a three-track network to simultaneously process MSAs, residue pair distances, and 3D coordinates. RoseTTAFold achieves a comparable accuracy to AF2 while offering great flexibility in some settings. Variants such as ColabFold [70] provide an optimized pipeline that reduces computational costs with minimal loss in accuracy. OpenFold [71] provides an open-source reimplementation of AF2 with a comparable accuracy but a faster speed and lower memory usage, while SPIRED [72] can address high-throughput demands by delivering significant speed improvements and reduced computational costs while achieving a performance comparable to OmegaFold [73]. AFsample2 [74] introduces stochastic masking of MSA columns to attenuate co-evolutionary constraints, thereby enhancing the structural diversity in models generated by AF2. More recently, Zheng et al. developed D-I-TASSER [75], which combines deep learning with classical physics-based folding simulations to tackle multidomain proteins. Benchmarking and CASP15 results show that D-I-TASSER outperforms both AF2 and AF3 on single and multidomain targets. Despite these advances, the models mentioned above primarily depend on MSAs to extract coevolutionary signals and guide structure predictions, rendering them ineffective for rapidly evolving proteins or wholly synthetic sequences that lack sufficient homologous data. To address this limitation, pre-trained protein language models (PLMs) like OmegaFold [73] and ESMfold [29] learn evolutionary information patterns directly from large quantities of raw protein sequences, removing the requirement for MSAs. These PLM-based models enable faster, single-sequence structure inference and are particularly valuable for orphan, rapidly evolving genes or synthetic sequences. However, benchmarking studies reveal a clear trade-off: PLMs can be competitive in some low-MSA-depth scenarios, even approaching or exceeding MSA-based performance, but when deep MSAs or strong template information are available, MSA-dependent models generally exhibit a higher accuracy [37,76]. Practically, de novo design workflows therefore often feature a tiered strategy: PLM/MSA-free predictors are used for a high-throughput, first-pass screening of novel sequences and orphan designs, and MSA-dependent models (or rescoring with high-confidence structure models) are applied when homologous data or templates exist and atomic detail is required to increase confidence in foldability and function [77].

Biological function is frequently mediated by biomolecular complexes or macromolecular assemblies consisting of proteins, nucleic acids, and small molecules. Consequently, beyond predicting the structures of single proteins, AI-driven models are increasingly capable of predicting complex multi-component assemblies [78]. AlphaFold-Multimer [79] extends the AF2 architecture to accurately predict the structures of multi-chain protein complexes. RoseTTAFoldNA [80] further broadens this capability by expanding the scope of structural prediction to include interactions between proteins and nucleic acids. RoseTTAFold All-Atom [60] and AF3 [56] have enhanced the capabilities of modeling comprehensive biological molecular systems through unified frameworks, enabling accurate structure predictions for complexes containing proteins, small molecules, nucleic acids, ions, and post-translational modifications. These breakthroughs provide a more comprehensive understanding of biological activities in diverse macromolecular systems. Building on AF3, Chai-1 [81] incorporates protein language model embeddings and advanced structural constraints, yielding greater flexibility and precision in predicting biomolecular assemblies. Boltz-1 [82] enhances the core AF3 architecture with targeted optimizations to boost accuracy and computational efficiency for multi-component assemblies. Building upon this framework, Boltz-2 [83] not only predicts the structure of protein–biomolecule interactions but also quantitatively estimates their binding affinity.

Advances in AI-driven structure prediction have driven the progression from accurate models of single protein structures to complex multi-component assemblies and dynamic conformational ensembles [84,85]. When integrated with downstream design algorithms, they form a unified workflow for de novo engineering: providing in silico validation and enabling rapid virtual screening of designed sequences and complexes to evaluate structural integrity, binding site fidelity, and interface compatibility, and thereby significantly reducing the experimental burden. Moreover, by combining high-throughput structure prediction with clustering methods such as Foldseek [86], researchers can systematically survey billions of candidate models, differentiate genuinely new structures from those cataloged in the Protein Data Bank (PDB), and validate new protein architectures with potential functional innovations across structure space [25].

### 4.2. De Novo Backbone Generation

In de novo protein design, generating novel folds that transcend evolutionary and natural templates is critical for accessing the protein structure space. While structure prediction models can accurately model proteins given a sequence, they are not designed to sample diverse backbone ensembles under tailored biophysical constraints [67]. Conversely, physics-based design engines like Rosetta rely on global energy minimization and therefore inherently limit both the scaffold diversity and design flexibility [39].

Diffusion models overcome these limitations by learning complex structural distributions directly from data, enabling the generation of highly diverse and precise backbone geometries while allowing the incorporation of custom constraints during training [87]. The Baker laboratory’s RFdiffusion [88] (a denoising diffusion probabilistic model fine-tuned from RoseTTAFold) has emerged as a benchmark for de novo backbone generation, supporting both unconditional scaffold generation and topology-constrained design across diverse applications. RFdiffusion [88] has demonstrated efficacy in designing high-affinity binders, assembling symmetric oligomers, and constructing scaffolds and functional motifs. Building upon this core framework, subsequent extensions of RFdiffusion address a spectrum of design challenges: RFdiffusion All-Atom [60] integrates atomic-level geometric constraints and explicit small-molecule binding specifications to generate protein pockets with tailored ligand affinities; RFantibody [89] adapts the model for de novo nanobody scaffold design; RFdiffusion-IDP Binder [90] and RFdiffusion β-Strand Binder [91], respectively, generate binders targeting intrinsically disordered regions and complement irregular β-sheet features (e.g., twists, bends, and bulges); RFpeptides [92] enhances the generation of macrocyclic peptides with defined topologies, and most recently, RFdiffusion2 [93] enables the direct scaffolding of atomically defined enzyme active sites without the need to pre-specify residue indices or enumerate side-chain rotamers, representing a significant advance in computational enzyme engineering. Furthermore, FrameDiff [94] implements diffusion-based generation without reliance on pretrained prediction networks, thereby potentially avoiding biases toward known natural structures.

Chroma [95] features a programmable generative process that integrates biophysically informed diffusion mechanisms with quasi-linear graph neural networks, enabling constraint-guided protein structure generation based on geometric constraints, symmetry, topology, and semantic prompts. ROS [96] employs a hallucination-based protein design strategy that operates within a relaxed sequence space [97,98], outperforming existing methods such as RFdiffusion in the design of large proteins (>600 amino acids). Flow-matching approaches eliminate the need to simulate stepwise diffusion during training, greatly reducing the computational cost for high-dimensional data and improving the training efficiency. Proteína [99] leverages flow matching for protein backbone generation and employs a non-equivariant Transformer architecture with 400 million parameters (five times larger than RFdiffusion) and can generate protein structures up to 800 amino acids in length.

### 4.3. “Fixed-Backbone” Sequence Design

After generating novel backbones, the core challenge in de novo protein design becomes inverse folding—identifying amino acid sequences that will reliably fold into a specified backbone while achieving the desired function. A standard metric for evaluating inverse-folding methods is the natural sequence recovery (NSR) rate, defined as the fraction of designed residues that match the native sequence of a naturally occurring protein with a similar fold. NSR is a widely used benchmark for assessing inverse folding models and has been widely used to compare methods. However, it has important limitations [100]. Inverse folding is inherently one-to-many: distinct, non-homologous sequences can stabilize the same backbone through alternative packing and compensatory substitutions, so strict recovery of a single “native” sequence can restrict novel yet biophysically valid solutions. Moreover, natural sequences encode evolutionary constraints (expression, regulation, promiscuous interactions, cellular context, immune selection, etc.) that may not align with specific engineering objectives (for example, enhanced thermostability or bespoke binding specificity); consequently, high NSR does not guarantee the intended functional phenotype. Most current inverse folding models also report structure-prediction metrics for the designed sequences (e.g., RMSD and pLDDT/pTM) and sequence novelty as a reference; we recommend, where possible, validating candidate sequences with task-relevant experimental assays (expression, solubility/stability, binding affinity, or deep mutational scanning).

ESM-IF [101] leverages a hybrid architecture combining protein language and structural modeling to generate proteins with substantially divergent sequences from natural evolutionary distributions. Furthermore, developed by the Baker laboratory, ProteinMPNN [102] has become a broadly adopted inverse-folding model. It implements a graph-based message-passing neural network (MPNN) architecture that includes a three-layer equivariant encoder to embed backbone atoms (N, C, O, Cα, Cβ) as a distance-weighted graph and a sequence-agnostic stochastic decoder to sample optimal amino acids at each position, producing diverse, topology-informed designs. It achieved a notable NSR of 52.4% for a test set of 402 monomer backbones (compared with Rosetta’s 32.9%) and has been experimentally validated, showing that its designed sequences reliably fold as intended [103,104]. LigandMPNN [105] extends the ProteinMPNN [102] framework and further incorporates non-protein ligands such as small molecules, nucleic acids, and metal ions into the design process, enabling optimization of protein ligand interfaces. Inspired by AlphaFold, CarbonDesign [106] employs an “Inverseformer” network that integrates multimodal constraints from structural features and ESM2-derived evolutionary embeddings. This architecture delivers breakthroughs in designing long-chain and novel proteins, outperforming established methods such as ProteinMPNN [102] and ESM-IF [101] across independent benchmarks, including CAMEO and CASP15, and de novo backbones generated by RFdiffusion [88]. CARBonARa [107] further enhances inverse folding by jointly modeling atomic coordinates and molecular environment constraints, significantly improving sequence-prediction accuracy in functional interface regions.

The capacity to engineer proteins with precise structural constraints is a prerequisite for the rational design of novel proteins with tailored functions. De novo backbone generation models overcome the fixed-topology limitations of classical design methods by systematically sampling the entire protein structure space, thereby uncovering previously inaccessible structures. In parallel, inverse folding algorithms identify amino acid sequences that not only adopt these specific backbones but also confer the desired functions. This synergistic, two-stage design strategy broadens the exploration of the sequence and structure space and, by extension, the functional universe, establishing an end-to-end pipeline from functional objective to tailored structure to sequence realization.

### 4.4. Sequence Generation

Recent advances in large language models (LLMs) have propelled the development of sequence generation models for de novo protein design, including ProtGPT2 [108], ProGen [109], ProGen2 [110], and TourSynbio [111]. These protein language models (PLMs) are pre-trained on millions of natural protein sequences, and treat each sequence as a “sentence” and each amino acid as a “token”. PLMs can generate novel sequences that conform to the learned biophysical and thermodynamic “grammar” of natural proteins, and can be fine-tuned for specific tasks such as enzyme or binder design [52]. However, the ability of PLMs to produce truly novel, broadly useful sequences is constrained by biases in their training data. Public sequence resources and curated databases are taxonomically and experimentally uneven (over-representing model organisms, pathogen- and human-related sequences, and protein families that are easy to assay), while structural and functional annotation lags far behind raw sequence growth. These sampling biases influence model likelihoods and sampling behavior, tending to push generation toward well-represented motifs and taxa rather than underexplored regions of the protein space [112,113]. To mitigate such artifacts, groups commonly augment or reweight the training corpus with metagenomic sequences and apply controlled fine-tuning on target design families; combined with structure- and energy-based rescoring and targeted experimental validation, these steps increase the likelihood that generated sequences are both novel and functional [109,114]. ProGen [109] is a 1.2 billion parameter neural network trained on 280 million sequences from over 19,000 annotated families that does not require structural data, while ProGen3 [27] scales this approach to 46 billion parameters pre-trained on 1.5 trillion amino-acid tokens drawn from 3.4 billion full-length proteins. Meanwhile, xTrimoPGLM [115] is a protein general language model that employs a dual objective training scheme using 100 billion parameters and 1 trillion tokens to jointly optimize protein understanding and design. It was pre-trained on 940 million unique protein sequences, comprising roughly 200 billion residues.

Protein sequences encode the blueprint for both protein structure and function [116]. Sequence generation models are trained on billion-scale protein databases, treating amino acid chains as a “language” whose grammar reflects the intrinsic evolutionary patterns and sequence features of protein structures. By leveraging this protein “grammar” to explore the sequence space and generate novel sequences, these models in turn drive the discovery and expansion of the protein functional universe.

### 4.5. Sequence–Structure Co-Design

Most de novo design methods typically generate sequences or structures separately, limiting their ability to capture the complex, bidirectional dependencies between them that govern protein folding functions [117]. Sequence–structure co-generation methods address this gap by jointly modeling both modalities, combining structure and sequence-driven insights to explore new regions of the functional universe. Several co-generation frameworks have recently emerged, including Multiflow [118], ProteinGenerator [119], ESM3 [120], and Pinal [121]. ProteinGenerator [119] is a RoseTTAFold-based sequence-space diffusion model that generates sequence–structure pairs through iterative denoising guided by specified sequence and structural attributes. ESM3 [120] is a frontier multimodal generative language model with 98 billion parameters, trained on 2.78 billion protein sequences and 771 billion unique tokens. It can jointly reason on protein sequences, structure, and function, yielding improved representation and generative evaluation across all three modalities. Pinal [121] employs a two-stage pipeline in which a natural language functional description is first converted into structural constraints and then conditioned sequences are generated on both the description and the resulting structure, achieving higher quality protein designs by operating within a smaller structural space.

By integrating sequence and structure within a unified generative framework, co-generation methods overcome the intrinsic limitations of two-stage generative design approaches; information flows unidirectionally—first through the generation of backbones and then through mapping them to sequences—hindering a full traversal of the sequence-structure-function landscape. Likewise, sequence generation methods neglect the critical structural constraints that underpin protein function. These separations fail to capture bidirectional dependencies and often lead to suboptimal designs. In contrast, sequence–structure co-generation enables coordinated exploration of the vast sequence–structure space, revealing previously inaccessible regions of the protein functional universe and directly yielding novel proteins with defined folds and tailored activities.

**Table 2:** Classification and Key-Attribute Comparison of AI-driven de novo Protein Design Tools.

Model Name	Release Date	Experimental Validation	Model Description	Algorithms	Efficiency	Advantage	Limitation	Is the Code Publicly Available?	Ref.
Protein Structure Prediction									
Alphafold2	15 July 2021	/	The first model predicts protein structures with atomic accuracy.	Evoformer	Low	Atomic accuracy	Resource-intensive; MSA-dependent	https://github.com/google-deepmind/alphafold (accessed on 5 September 2025)	[67]
RoseTTAFold	19 August 2021	/	Accurately predicts protein structures and interactions.	3D Transformer	Middle	Fast, flexible; accuracy	Less accurate than AF2 on hard targets	https://github.com/RosettaCommons/RoseTTAFold (accessed on 5 September 2025)	[69]
ColabFold	30 May 2022	/	Fast and easy for the prediction of protein structures and complexes.	Evoformer	High	Fast, accessible	Depends on the AF2 back end	https://github.com/sokrypton/ColabFold (accessed on 5 September 2025)	[70]
OmegaFold	2 July 2022	/	Predict orphan proteins and rapidly evolving antibodies.	PLM	High	Fast, MSA-free	Slightly lower accuracy on some targets	https://github.com/HeliXonProtein/OmegaFold (accessed on 5 September 2025)	[73]
ESMFold	16 March 2023	/	Predict protein structure with atomic precision using language models.	PLM	High	Fast, MSA-free	Lower atomic precision vs. AF2	https://github.com/mit-ll/ESMFold (accessed on 5 September 2025)	[29]
OpenFold	14 May 2024	/	An open-source, trainable implementation of AF2.	Evoformer	Middle	Open, reproducible AF2 implementation	Similar computational needs to AF2	https://github.com/aqlaboratory/openfold (accessed on 5 September 2025)	[71]
SPIRED	27 August 2024	/	Enhances prediction speed, reduces training consumption.	Unit-based	Middle	Faster training/inference; efficient design	Accuracy below AF2	https://github.com/Gonglab-THU/SPIRED-Fitness (accessed on 5 September 2025)	[72]
AFsample2	5 March 2025	/	Expands the structural diversity of AF2’s generative models.	Stochastic sampling	Low	Produces conformational ensembles	Much higher computation for sampling	https://github.com/iamysk/AFsample2 (accessed on 5 September 2025)	[74]
D-I-TASSER	23 May 2025	/	Predicts large multidomain protein structures.	Deep and physics	Low	Good for multi-domain proteins	Slow; template/MSA-dependent	https://zhanggroup.org/D-I-TASSER/download/ (accessed on 5 September 2025)	[75]
Predicted multimers structure									
AlphaFold Multimer	10 October 2021	/	Predicts the structure of protein complexes.	Evoformer	Low	Improved multimer predictions	Higher computation requirements	https://github.com/jcheongs/alphafold-multimer (accessed on 5 September 2025)	[79]
RoseTTAFoldNA	23 November 2023	/	Predicts the structures of protein-nucleic acid complexes.	3D Transformer	Middle	Predicts protein–nucleic acid complexes	Limited NA training data	https://github.com/uw-ipd/RoseTTAFold2NA (accessed on 5 September 2025)	[80]
RoseTTAFold All-Atom	7 March 2024	/	Predicts the structures of biomolecular assemblies containing proteins, nucleic acids, small molecules, metals, and chemical modifications.	3D Transformer	Low	Models small molecules/ions	Computationally demanding, high memory	https://github.com/baker-laboratory/RoseTTAFold-All-Atom (accessed on 5 September 2025)	[60]
AlphaFold3	8 May 2024	/	Predicts biomolecular complexes such as proteins, nucleic acids, small molecules, ions, and modified residues.	Evoformer	Low	Multimolecule unified modeling	Large computational requirements	https://github.com/google-deepmind/alphafold3 (accessed on 5 September 2025)	[56]
Chai-1	11 October 2024	/	Predicts the structures of protein–ligand complexes and protein multimer.	PLM	High	Aims MSA-free multimodal prediction	Public details limited	https://github.com/chaidiscovery/chai-lab (accessed on 5 September 2025)	[81]
Boltz-1	20 November 2024	/	Open-source AF3-level precision prediction model.	Evoformer	High	Optimized AF3 architecture	Public details limited	https://github.com/jwohlwend/boltz (accessed on 5 September 2025)	[82]
Boltz-2	6 June 2025	/	Simultaneous prediction of protein–small molecule complex structures and binding affinity.	Evoformer	High	Adds affinity estimation to the structure	Public details limited	https://github.com/jwohlwend/boltz (accessed on 5 September 2025)	[83]
De novo protein backbone generation									
RFdiffusion	11 July 2023	Yes	Unconditional/topology monomers; binders; symmetric oligomers; enzyme scaffolds; motif scaffolds.	Diffusion	Middle	Versatile backbone generation	Sampling is computationally allyintensive	https://github.com/RosettaCommons/RFdiffusion (accessed on 5 September 2025)	[88]
FrameDiff	22 May 2023	No	Independent monomer generation with up to 500 amino acids without pretraining.	Diffusion	High	Efficient; no pretrained predictor needed	Needs more validation	https://github.com/jasonkyuyim/se3_diffusion (accessed on 5 September 2025)	[94]
Chroma	15 November 2023	Yes	Programmable protein generation via symmetry, shape, class, or text inputs.	Diffusion	High	Efficient, scalable	Limited benchmarks	https://github.com/generatebio/chroma (accessed on 5 September 2025)	[95]
FoldingDiff	5 February 2024	No	Unconditionally generates highly realistic protein structures.	Diffusion	High	Scales to long chains	Indirect side-chain generation	https://github.com/microsoft/foldingdiff (accessed on 5 September 2025)	[122]
RFdiffusion All-Atom	7 March 2024	Yes	Ligand-guided de novo protein scaffold design.	Diffusion	Middle	Atomistic pockets and ligand design	Computationally costly	https://github.com/baker-laboratory/rf_diffusion_all_atom (accessed on 5 September 2025)	[60]
RSO	24 October 2024	Yes	Relaxed-sequence optimization enabling large-scale protein design without retraining.	Hallucination-based	Low	Joint sequence/structure optimization	Local optimum risk	https://github.com/sokrypton/ColabDesign (accessed on 5 September 2025)	[96]
SCUBA-D	21 November 2024	Yes	Unconditional generation; generation based on sketch input; motif scaffolding.	Diffusion	Middle	Sample novel folds	Experimental validation needed	https://github.com/liuyf020419/SCUBA-D (accessed on 5 September 2025)	[123]
Proteina	2 March 2025	No	Unconditional/class-conditional generation; motif scaffolding.	Flow-matching	High	Generates long chains (up to 800 aa)	Needs side-chain step	https://github.com/NVIDIA-Digital-Bio/proteina (accessed on 5 September 2025)	[99]
RFdiffusion2	10 April 2025	Yes	Atom-level active site scaffolding without residue indexing or rotamer sampling.	Diffusion	Middle	Direct active-site scaffolding	Computationally costly	https://github.com/RosettaCommons/RFdiffusion2 (accessed on 5 September 2025)	[93]
ProtComposer	6 March 2025	No	Ellipsoid-guided protein generation with customizable layouts.	Flow-matching	High	Conditional layout control	Complex implementation	https://github.com/NVlabs/protcomposer (accessed on 5 September 2025)	[124]
TopoDiff	18 June 2025	Yes	Enabling both unconditional and controllable diffusion-based protein generation.	Diffusion	High	Topology-controlled design	Public details sparse	https://github.com/meneshail/TopoDiff/tree/main (accessed on 5 September 2025)	[125]
‘Fixed-backbone’ sequence design									
ESM-IF	10 April 2022	Yes	Inverse folding (protein complexes, partially masked structures, binding interfaces, and multiple states).	PLM	Middle	PLM priors improve novelty	Only backbone design	https://github.com/facebookresearch/esm (accessed on 5 September 2025)	[101]
ProteinMPNN	15 September 2022	Yes	Inverse folding (monomers, cyclic oligomers, protein nanoparticles, and protein-protein interfaces).	MPNN	High	Fast; strong inverse-folding performance	Ignores the ligand context	https://github.com/dauparas/ProteinMPNN (accessed on 5 September 2025)	[102]
ProRefiner	16 November 2023	Yes	Structure-guided residue sequence inpainting with entropy-based global noise filtering.	Transformer	Middle	Improves model outputs	Training complexity	https://github.com/veghen/ProRefiner (accessed on 5 September 2025)	[126]
CarbonDesign	23 May 2024	No	Inverse folding, zero-shot prediction of mutational effects on protein function.	Transformer	Middle	Multimodal constraint integration	Needs broader benchmarking	https://github.com/carbon-design-system/carbon (accessed on 5 September 2025)	[106]
CARBonAra	25 July 2024	Yes	Designs protein sequences under the constraints of specific molecular interaction environments.	Transformer	Middle	Handles ligand/metal contexts	Training complexity	https://github.com/LBM-EPFL/CARBonARa (accessed on 5 September 2025)	[107]
LigandMPNN	28 March 2025	Yes	Simultaneously outputs ligand-binding sequences and sidechain conformations for interaction analysis.	MPNN	Middle	Designs ligand interfaces	Requires ligand coordinates	https://github.com/dauparas/LigandMPNN (accessed on 5 September 2025)	[105]
FAMPNN	17 February 2025	No	Full-atom protein sequence design.	MPNN	High	Full-atom sequence and sidechain output	Limited public details	https://github.com/richardshuai/fampnn (accessed on 5 September 2025)	[127]
Methods generating sequences									
ProtGPT2	27 July 2022	No	High-throughput de novo protein sequence generation.	Transformer	High	Fast generation	Limited control	https://github.com/TeletcheaLab/protGPT2 (accessed on 5 September 2025)	[108]
ProGen	26 January 2023	Yes	Generates functional artificial proteins across families based on a conditional language model.	Transformer	Middle	Conditional generation possible	Computationally demanding	https://github.com/salesforce/progen (accessed on 5 September 2025)	[109]
ESM2	16 May 2023	Yes	Learns evolutionary patterns for accurate structure–function prediction.	PLM	High	Excellent embeddings; fast	Not primarily generative	https://github.com/facebookresearch/esm (accessed on 5 September 2025)	[29]
ProGen2	15 November 2023	No	Evolutionary modeling, de novo generation, and zero-shot fitness prediction.	Transformer	Low	Strong generative power	Resource heavy	https://github.com/anonymized-research/progen2 (accessed on 5 September 2025)	[110]
xTrimoPGLM	3 April 2025	No	Large-scale language models for protein analysis and design.	Hybrid	Low	Scales to large tokens	Training/inference costly	https://github.com/ONERAI/xTrimoPGLM (accessed on 5 September 2025)	[115]
ProGen3	16 April 2025	Yes	Its scale enables broader viable protein generation.	Transformer	Low	Super-scale generative model	Extremely resource-intensive	https://github.com/Profluent-AI/progen3 (accessed on 5 September 2025)	[27]
Sequence–structure co-design									
Multiflow	7 February 2024	No	DFMs and multiflow enable protein co-design.	Flow-matching	High	Accurate joint sequence–structure	No side-chain output	https://github.com/jasonkyuyim/multiflow (accessed on 5 September 2025)	[118]
ProteinGenerator	25 September 2024	Yes	Generates diverse de novo proteins under customizable sequence constraints.	Diffusion	Middle	Property-guided seq–structure co-design	Weak on long protein	https://github.com/RosettaCommons/protein_generator (accessed on 5 September 2025)	[119]
ESM3	16 January 2025	Yes	Supports multi-modal prompt control (sequences, structures, and functions) for generating proteins.	PLM	Low	Multimodal reasoning	Computationally demanding	https://github.com/Cogibra/esm3 (accessed on 5 September 2025)	[120]
Pinal	31 March 2025	No	Protein structure and language co-constrained sequence design.	Transformer	Low	Text → structure → sequence pipeline	Limited by training distribution biases	https://github.com/westlake-repl/Denovo-Pinal (accessed on 5 September 2025)	[121]

## 5. AI as an Engine for Protein Functional Universe Exploration

**Figure 4:**
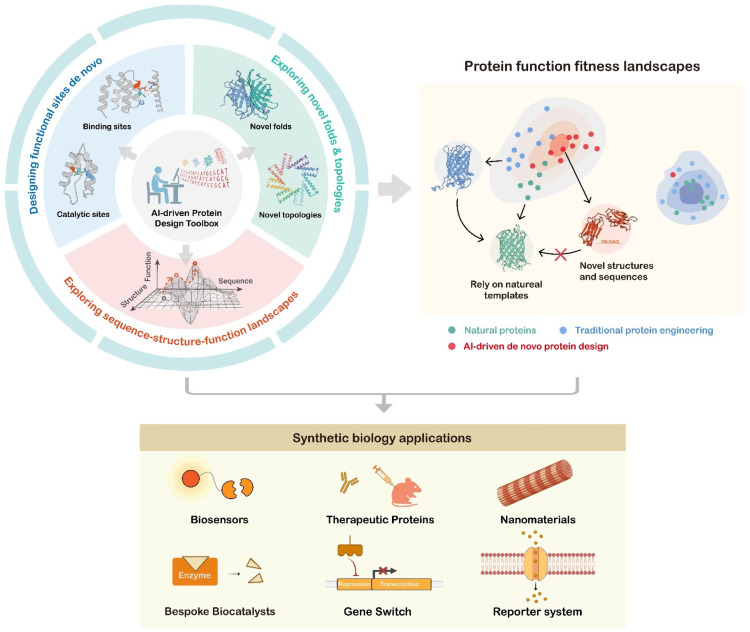
AI-driven de novo protein design can explore the structure space to uncover novel folds and topologies, engineer bespoke functional sites for defined activities, and thereby perform the global exploration of the sequence–structure–function landscape. Free from evolutionary constraints, it enables access to entirely new protein functions and more readily reaches global fitness optima—innovations that have already powered diverse synthetic biology applications.

AI is rapidly emerging as the principal driver for systematic protein functional exploration by incorporating accurate structure prediction, de novo design, and iterative experimental feedback into a seamless discovery engine. Generative frameworks now propose novel fold topologies and candidate sequences that expand the accessible sequence–structure space, while predictive models trained on biochemical and phenotypic assays provide rapid in silico triaging of catalytic efficiencies, binding affinity, and other functional indicators [128,129,130,131,132]. These components are commonly integrated into a closed-loop “design–predict–test–learn” workflow: (i) an acquisition policy (often based on a predicted score, uncertainty, or an acquisition function) selects a small, high-value set of candidates for synthesis and assay; (ii) experimental readouts (e.g., binding K_D_, k_cat_/K_m_, stability, expression) are quality-controlled and preprocessed to account for assay noise and batch effects; and (iii) the resulting labeled data are used to update models via targeted fine-tuning, retraining of surrogate predictors, or Bayesian/active-learning schemes that prioritize the next experiments. In practice, effective feedback requires uncertainty-aware models (to avoid overconfident selection), multi-fidelity modeling, and methods for domain adaptation when designs depart from the training distribution. Major challenges remain—the limited experimental throughput and cost, inconsistent assays, label sparsity for rare functions, and potential distribution shifts between training data and designed sequences—and these practical constraints shape algorithmic choices. Automation (robotics, standardized metadata, and data pipelines) and careful reporting of model scores and assay conditions improve the speed and reliability of the loop, but exploration must still be balanced with pragmatic developability and biosafety considerations. Through this seamless cycle, AI transforms de novo protein design from a laborious trial-and-error process into a rational, guided, data-centric exploration, unlocking completely new biochemical transformations, substrate specificities, and regulatory mechanisms within the protein functional universe and accelerating the development of next-generation therapeutic proteins [133,134], biocatalysts [93,135,136], biosensors [137,138], and self-assembling materials [139,140]. This underpins the assembly of ever more sophisticated synthetic biology circuits for precise cellular regulation (Figure 4).

### 5.1. Exploring Novel Folds and Topologies

Yeo et al. clustered 821 million structures from AFDB and ESMatlas by sequence and structural similarity, yielding 5.12 million non-singleton clusters. Strikingly, despite encompassing over 600 million predicted structures, ESMatlas yielded only one novel fold when compared to the more than 200 million entries in AFDB, representing only a minute fraction of the theoretical landscape of possible protein folds and underscoring that vast regions of the structure space remain unexplored [25]. AI-driven methods have begun to fill these gaps by uncovering entirely new structures. RFdiffusion All-Atom produced functional small-molecule binders for digoxigenin, heme, and bilin that adopt non-natural backbone geometries with a near-zero sequence homology to any PDB entry yet achieve high-affinity binding via unprecedented folds [60]. Similarly, SCUBA-D’s unconditional sampling of just 500 backbones revealed multiple topologies absent from nature, confirming that vast uncharted regions persist within the structure space [123]. TopoDiff further expanded this space by designing proteins composed exclusively of β strands and coils—none of which resemble known structures [125]. AI methods also enable the creation of modular twistless helix-repeat (THR) models that assemble similar molecular “Lego” to form polygons, rings, cages, and tubes of a defined size [139]. These results demonstrate that AI methods can systematically explore and experimentally realize protein structures far beyond the limits of natural evolution.

### 5.2. Designing Functional Sites De Novo

AI-driven de novo protein design achieves functional diversification through de novo construction of functional sites such as tailored binding pockets, catalytic centers, and allosteric regulators—transforming the function-first design blueprint from concept to reality.

For instance, Wu et al. integrated physics-based and deep-learning approaches (e.g., RFdiffusion) to build binding pockets for intrinsically disordered-region sequences. Screening 22 designs per target across 39 unstructured targets, they observed binders with affinities of 100 pM-100 nM for 34 targets. Glögl et al. then tackled the challenge of flat, polar interfaces by designing TNFR1 antagonists (Kd < 10 pM) that effectively inhibit TNF-α signaling and demonstrate efficacy against inflammatory cascades previously resistant to inhibition [133]. Beyond the binding pocket, Pillai et al. created switchable assemblies whose oligomeric state transitions in response to small-molecule effectors, enabling allosteric control systems for drug delivery and dynamic regulation of synthetic cellular pathways [140]. On the catalytic site design front, Hou et al. developed a class of artificial protein catalysts termed NovoChromes, which bind both heme and synthetic porphyrins to efficiently catalyze nonnatural reactions, including cyclopropanation and silylation. Through de novo design and directed evolution, these catalysts achieve high efficiency and stereoselectivity, exhibiting remarkable stability in concentrated organic solvent conditions (up to 70% ethanol) and under high thermal resistance (Tm > 90 °C) [141].

Together, these examples illustrate how AI tools, by specifying only the desired function, can autonomously sculpt atomically precise functional geometries within synthetic frameworks, unlocking previously inaccessible regions of the protein functional universe.

### 5.3. Exploring Sequence–Structure–Function Landscapes

Proteins exist in a high-dimensional sequence–structure–function landscape whose topology is extremely rugged. Each protein sequence corresponds to a point defined by its three-dimensional structure and its functional properties. Due to pervasive epistasis (mutations interacting in non-additive ways), the fitness landscape is pockmarked with many local optima [142,143]. In practical terms, this means that changing one amino acid can have very different effects depending on the rest of the sequence; thus, evolutionary or traditional protein engineering searches often become trapped in local peaks and fail to find globally optimal solutions. Most computational design methods must “navigate a rugged fitness landscape incrementally”, much like natural evolution does [95,144]. Directed evolution or physics-based design thus tends to explore only a small sub-region of the sequence space.

By contrast, modern AI approaches learn continuous latent embeddings of protein sequences and structures that capture underlying biophysical and evolutionary constraints [120]. Within these latent spaces, AI models can propose novel sequences and topological structures and direct probabilistic sampling toward regions of higher predicted fitness, thereby efficiently navigating the complex sequence–structure–function landscape [119].

AI-driven de novo protein design has already explored novel regions of protein function that nature never sampled. For example, a multimodal language model ESM3 that considers sequence, structure, and function was prompted to generate fluorescent proteins. Among the designs was “esmGFP”, a bright green fluorescent protein only 58% identical to any known example, placing it in a region of the functional landscape that natural evolution never explored [120]. Similarly, Gao et al. introduced AiCE, a framework that samples from inverse-folding models with structural and evolutionary constraints. AiCE tackled eight distinct engineering challenges—covering deaminase enzymes, nucleases, base editors, and more—achieving success rates between 11% and 88% in identifying improved variants and efficiently navigating the fitness landscape across multiple structures and functions [145].

In addition, AI-driven de novo protein design still depends on the integration of experimental feedback; as Biswas et al. showed, latent models guided only by evolutionary priors tend to avoid nonfunctional regions in fitness landscapes. In addition, without any real experimental data, they may not find the highest-activity variants [38].

AI-driven de novo protein design continually sharpens our view of the sequence–structure–function landscape and empowers us to move beyond incremental tweaks. By iterating between AI-driven exploration and wet-lab validation, initial models can delineate the boundaries of promising sequence regions, with targeted lab validation filling in the gaps and correcting biases. This feedback loop refines the latent embeddings, making the model’s landscape smoother and more accurate around high-fitness areas [146]. We can progressively reveal and exploit the global peaks of protein functionality to not only overcome local traps in the sequence–structure–function landscape but also to steadily expand the frontiers of the protein functional universe.

### 5.4. AI-Driven De Novo Protein Design for Applications in Biotechnology and Synthetic Biology

AI-driven de novo protein design is rapidly maturing from a method-focused discipline into a practical engine for biotechnology and synthetic biology. In this Section 5.4, we summarize representative application domains—therapeutic proteins, enzyme engineering, biosensors/materials, and synthetic biology regulators—and summarize design objectives and computational workflows, together with developability and performance metrics (Table 3).

In therapeutics, target/epitope specification has led to the production of compact proteins and peptides with high affinity, exceptional stability, and demonstrable efficacy in cellular and animal models. Vázquez Torres et al. engineered miniproteins that neutralize snake venom toxins, achieving 100% survival in envenomed mice with exceptional thermal stability (Tm > 95 °C) and nanomolar affinity [134]. Mahling et al. used Colabdesign [147] to produce a 21-residue peptide (ELIXIR) that selectively inhibits pathological late or persistent Na^+^ current (*I*_NaL_) by enhancing Na_V_1.5 channel inactivation. ELIXIR binds to the Na_V_1.5 C-terminal domain with K_D_ = 0.89 ± 0.25 μM, achieving > 90% *I*_NaL_ inhibition in the disease models tested. Functionally, ELIXIR restores elevated *I*_NaL_ toward healthy levels in patient-derived iPSC cardiomyocytes and markedly reduces *I*_NaL_ and shortens QTc in transgenic mouse models [148].

In enzyme engineering, Listov et al. developed a computational enzyme-design workflow that produced efficient Kemp eliminase without further experimental optimization. Of the 73 designs tested, 3 (~4.1%) exhibited measurable Kemp-elimination activities. The top variant, Des27.7, achieved k_cat_/K_m_ = 1.27 × 10^4^ M^−1^s^−1^, an approximately 60-fold improvement over the initial design; the experimentally observed active-site conformation matched the design model to within 0.5 Å [149]. Lauko et al. employed a deep learning-driven approach to de novo enzyme design, resulting in a serine hydrolase with a previously unobserved protein fold. The designed enzyme demonstrated a catalytic efficiency (k_cat_/K_m_) of up to 2.2 × 10^5^ M^−1^s^−1^. Among the 132 designed variants, 20% exhibited detectable hydrolytic activity [136]. Munsamy et al. used the ZymCTRL framework to design 20 carbonic anhydrase variants, each with <50% sequence identity to known enzymes; 7 of these (35%) exhibited measurable catalytic activity. Applying a similar design workflow to lactate dehydrogenase yielded 20 candidates, of which 14 (70%) displayed detectable enzymatic activity [150].

In synthetic biology, researchers aspire to “program” living systems by designing predictable genetic parts that drive cells to perform desired functions, and AI has emerged as a new enabling engine for this effort. Zhang et al. exemplified this approach by engineering intracellular Ras–GTP activity sensors (Ras-LOCKR-S) and proximity-labeling modules (Ras-LOCKR-PL) that operate with a subcellular spatial resolution; these tools were subsequently used to dissect mechanisms of resistance to Ras-G12C inhibitors [137,138].

**Table 3:** AI-driven de novo Protein Design—Biotech Applications Overview.

Molecule Number	Molecule	Target and Activity	Method	Indications/Function	Ref.
Therapeutic Proteins					
1 and 2 and 3	SHRT	Short-chain α-neurotoxins (ScNtx) K_D_ = 0.9 nM, Tm = 78 °C	RFdiffusion ProteinMPNN	Snake venom toxins.	[134]
	LNG	Long-chain α-neurotoxin (P01391) K_D_ = 1.9 nM, Tm > 95 °C			
	CYTX	Cytotoxins (Naja pallida) K_D_ = 271 nM, Tm = 61 °C			
4	TNFR1_mb2_pd1	The tumor necrosis factor receptor 1(TNFR1) K_D_ (TNFR1) < 10 pM	RFdiffusion ProteinMPNN	Inflammatory disease.	[133]
5 and 6 and 7	23R-91	Interleukin (IL)-23R K_D_ < 1 pM	Rosetta	Autoinflammatory diseases.	[48]
	17–53	IL-17 K_D_ = 10 pM			
8	ELIXIR	Na_V_1.5 carboxy-terminal domain K_D_ = 0.89 ± 0.25 μM	AfDesign	Cardiac arrhythmias and epilepsy.	[148]
Enzyme Engineering					
9	Serine hydrolases	k_cat_/K_m_ = 2.2 × 10^5^ M^−1^s^−1^	RFdiffusion LigandMPNN PLACER	Catalyze ester hydrolysis with catalytic.	[136]
10	Kemp eliminase	k_cat_/K_m_ = 1.27 × 10^4^ M^−1^s^−1^	Rosetta, PROSS FuncLib, AlphaFold2	Kemp elimination.	[149]
11	Metallohydrolases	k_cat_/K_m_ = 2.3 × 10^4^ M^−1^s^−1^	RFam, ProteinMPNN AlphaFold2	Catalyzes some difficult hydrolysis reactions.	[151]
12	Retroaldolase	k_cat_/K_m_ = 1.1 × 10^4^ M^−1^min^−1^	ChemNet, Rosetta LigandMPNN	Catalyze the reverse aldol reaction.	[152]
13 and 14	Carbonic anhydrases Lactate dehydrogenases	NA	ZymCTRL	The fastest enzymes known in nature. Primarily in lactic acid production.	[150]
Synthetic biological components					
15	Ras-LOCKR-S/PL	Ras-GTP	Rosetta, AlphaFold	Sensor for Ras activity. Ras activity-dependent Proximity Labeler.	[137]
16	THR	/	Rosetta, ProteinMPNN	Enables modular nanomaterial design.	[139]
17	Allosterically protein assemblies	/	Rosetta, ProteinMPNN, RFDiffusion AlphaFold2	Allosteric modulation.	[140]

## 6. Conclusions

AI-driven de novo protein design has substantially transformed our ability to explore the protein functional universe, enabling the rational engineering of novel folds, bespoke functional sites, and proteins with tailored biophysical properties—with a growing number of experimentally validated successes. To translate these advances into robust, scalable pipelines, we must address several practical challenges.

Model performance highly depends on the quality, diversity, and annotation of training data. Public sequence and structure resources are extensive but biased toward model organisms and readily assayed families, which limits generalization to under-represented families and wholly synthetic sequences. The experimental throughput, noisy or heterogeneous assays, and the distribution shift between training corpora and designed sequences further complicate the reliable translation from in silico designs to functional molecules. In addition, biosafety and validation must be integrated early: designs that introduce novel folds or activities can have unintended biological effects (toxicity, off-target interactions, or immune responses) and therefore require rigorous multiomic and phenotypic validation, together with explicit risk-mitigation strategies, prior to any in vivo work. Explicit risk assessment and traceable data sources are prerequisites for clinical or industrial applications [153,154,155].

Looking forward, models should be improved to better capture the multi-state conformational ensembles, allostery, and dynamic interactions that underpin biological function; they should also provide calibrated uncertainty estimates and more interpretable outputs to support users’ design choices. Progress will depend on improving dataset curation (for example, adding quality-filtered metagenomic sequences and richer functional labels), standardizing assay and reporting practices, and sharing community benchmarking datasets to increase robustness and comparability. We further recommend adopting unified scoring standards to enable direct, intuitive comparison among models. Finally, the computational infrastructure remains a key enabler. GPU-accelerated parallelism and optimized distributed training/inference stacks reduce iteration times and permit larger models and broader in silico screenings. Emerging quantum computing approaches may eventually assist specialized optimization [156,157,158].

In conclusion, AI has already opened new horizons for programmable biology. Realizing its full promise will require parallel progress on data, algorithms, computing, interpretability, and biological validation—combined with explicit biosafety safeguards—to ensure those horizons are explored responsibly and efficiently.

## Figures and Tables

**Table 1:** Quick workflows (Design goal → inputs → outputs summaries) for AI-driven de novo protein-design toolkits.

Toolkit	Goal	Inputs	Outputs
Protein structure prediction (Section 4.1)	Produce 3D models and confidence estimates for single-chain proteins or complexes to assess foldability and guide design.	One or more amino-acid sequences, optional partner sequences, ligands, oligomeric state, templates.	Predicted coordinates (PDB); per-residue and global confidence scores (pLDDT, pTM, PAE); interface metrics for complexes.
De novo backbone generation (Section 4.2)	Generate novel backbone geometries or scaffolds that satisfy specified geometric/functional constraints.	Design constraints (motif/active sites coordinates, desired topology/symmetry, pocket geometry, binder, anchor residues).	Ensemble of candidate backbone coordinates (atomic models).
‘Fixed-backbone’ sequence design (Section 4.3)	Design sequences that fold to a given backbone and meet developability.	Target backbone coordinates (PDB); optional side-chain/motif constraints.	Ranked sets of candidate sequences.
Sequence generation (Section 4.4)	Produce diverse candidate sequences de novo (unconditionally or conditionally guided).	Conditioning information (family or functional labels, motif, structural constraints, or descriptor prompts) and sampling parameters.	Batches of candidate amino-acid sequences annotated with model scores, novelty metrics and basic developability annotations.
Sequence–structure co-design (Section 4.5)	Jointly generate matched sequence–structure pairs that satisfy functional constraints.	Functional constraints (motif geometry, binding interface, text prompt).	Paired sequence–structure candidates.

## Data Availability

No new data were created or analyzed in this study.
